# #PRS: A Study of Plastic Surgery Trends With the Rise of Instagram

**DOI:** 10.1093/asjof/ojad004

**Published:** 2023-01-11

**Authors:** Kometh Thawanyarat, Chandler Hinson, Diego A Gomez, Mallory Rowley, Yelissa Navarro, Chandler Johnson, Chelsea M Venditto

**Affiliations:** Medical College of Georgia at Augusta University, Augusta, GA, USA; Frederick P. Whiddon College of Medicine, University of South Alabama, Mobile, AL, USA; Mayo Clinic Alix School of Medicine, Scottsdale, AZ, USA; State University of New York, Upstate Medical University, Syracuse, NY, USA; Medical College of Georgia at Augusta University, Augusta, GA, USA; Medical College of Georgia at Augusta University, Augusta, GA, USA; Medical College of Georgia at Augusta University, Augusta, GA, USA

## Abstract

**Background:**

Instagram (Menlo Park, CA) has become a popular means of advertisement for aesthetic surgery procedures, influencing patients’ likelihood of undergoing a procedure. In this study, the authors aim to explore public interest in aesthetic procedures before and after the Instagram platform started gaining in popularity through Google Trends (Google, Mountain View, CA), a platform with previously demonstrated utility for tracking interest in surgical procedures.

**Objectives:**

The authors hypothesize that as a result of increased medical marketing on Instagram, there is an increase in public interest in elective procedures of plastic surgery.

**Methods:**

Trends in the United States for given search terms and volumes were gathered through Google Trends between April 2004 and January 2022. Search terms included popular aesthetic procedures based on the 2020 Aesthetic Plastic Surgery National Data Bank Statistics. The search volumes were normalized, and a bivariate regression analysis of panel data was then applied to the aggregate trendlines to determine whether a statistically significant change in search volume occurred following the increase in user traffic of the Instagram platform.

**Results:**

The authors found significant variations in search volume for plastic surgery procedures before and after April 2012. Blepharoplasty, Botox, brachioplasty, breast implant removal, breast reduction, brow lift, butt lift, hair transplantation, lip augmentation, male breast surgery, mastopexy, mentoplasty, otoplasty, platysmaplasty, rhinoplasty, and thighplasty (*P* < .000) had statistically significant increases in search volume, whereas buccal fat removal (*P* = .003) had a statistically significant decrease in search volume after April 2012.

**Conclusions:**

The authors observed a significant increase in public interest in both surgical and nonsurgical aesthetic procedures after Instagram gained popularity in the April of 2012.

Instagram (Menlo Park, CA) has revolutionized the way society is interconnected. Since its founding in October 2012, Instagram has grown to over 1.4 billion users. The rapid growth of individual users on the platform created a channel for companies to directly market to their customers. As of February 2022, over 200 million companies have profiles on Instagram and actively interact with the general public. Within the field of plastic surgery, Instagram provides an opportunity for surgeons to create a brand and to visually demonstrate their surgical results to potential patients, allowing them to reach thousands with a single post.^1^

A unique example of this opportunity was the birth of the “Instagram Doll.” Instagram dolls are patients undergoing cosmetic surgery who document their experiences with a plastic surgeon on Instagram, providing their results for other patients or prospective clients.^2^ These individuals use hashtags to connect their work to the specific provider, creating a stream of potential clients directed toward the surgical practice. This represents 1 of the various ways that plastic surgeons are utilizing social media to reach a more expansive audience and gain a larger population of patients. Instagram in particular has shown to be 1 of the main tools used for plastic surgeon exposure, feedback, and connection within the plastic surgery realm.^3,4^

Outside of individual accounts, the use of hashtags has allowed for increased growth within certain realms of plastic surgery, allowing users to see the top-trending photographs based on a specific hashtag. In fact, patients commonly utilize hashtag searches to find plastic surgeons based on the photographs they identify. This allows Instagram to function as a 2-way platform: prospective patients can review aesthetic surgery results and previous patients can communicate their results, outcomes, and perspectives of the procedure.^5^ Essentially, Instagram can act as a powerful customer-review site, which can affect prospective patients’ desire to undergo aesthetic procedures with specific surgeons.

There is evidence that social media has led to an increase in the number of individuals who are seeking aesthetic surgery.^6^ The online search tool Google Trends (Google, Mountain View, CA) has become a common way of identifying temporal and geographic trends in online searches. Researchers in previous studies have employed this powerful tool to examine the public's interest in specific plastic surgery procedures, as well as interest in residency programs. For example, during the SARS-CoV-2 pandemic, there was an increase in the number of Instagram users desiring to learn about aesthetic procedures and their results. It has also been noted that the increased use of Zoom (San Jose, CA) calls during the pandemic led to an increase in the number of individuals interested in surgery, especially above-shoulder procedures.^7^ In this study, we seek to examine whether the release of Instagram and its subsequent popularity has correlated with increased public interest in elective cosmetic procedures.

## METHODS

Trends in the United States for given search terms and volumes were gathered through Google trends between April 1, 2004, and January 12, 2022, with the inflection date of April 1, 2012, marking the release of Instagram. The search volumes were normalized to facilitate comparisons between search terms. To normalize the data, the google trend tool compared the relative change in the trend of each term for a specific month and presented it as a proportion from the total searches within the United States. This volume was then scaled based on its popularity from 0 to 100 when compared with the other search terms. This resulted in search volumes compared among each search term as the listed value of popularity instead of absolute volumes that were not available on the google trend tool.

In this study, we included 25 aesthetically related surgical procedures in the United States from April 2004 to January 2022 (Table 1). The terms chosen were searched by their clinical terms as well as the procedures’ colloquial counterpart (ie, rhinoplasty and nose-job, respectively) using the 2021 Aesthetic Plastic Surgery National Data Bank Statistics.^8^Table 1 shows the terms grouped by procedure category to encompass all possible searches of the inquired procedures.

**Table 1.** Aesthetic Surgery Procedure Types and Associated Search Terms

Procedure	Search terms
Abdominoplasty	Abdominoplasty Tummy tuck Liposuction
Blepharoplasty	Blepharoplasty Eyelid plastic surgery Eyelid surgery
Botox	Botox Botox injection Botulinum toxin
Brachioplasty	Brachioplasty Upper arm lift Arm lift Arm lift surgery
Breast augmentation	Breast augmentation Breast augmentation surgery Breast implant surgery Boob job surgery Breast implant(s)
Breast implant removal	Breast implant removal Breast implant removal surgery Breast explantation
Breast reduction	Breast reduction Breast reduction surgery
Brow lift	Brow lift Forehead lift
Buccal fat removal	Buccal fat removal Facial fat transfer Facial fat grafting
Butt lift	Butt lift Brazilian butt lift Gluteal injection Butt injection Butt implant(s) Butt fat transfer Butt lift surgery
Calf augmentation	Calf augmentation Calf implants(s)
Hair transplant	Hair transplantation Hair restoration Hair transplant Hair transplant surgery
Lip augmentation	Lip augmentation Lip augmentation surgery Lip injection Lip fillers Lip surgery Lip enhancement
Lip reduction	Lip reduction Lip reduction surgery
Lower body lift	Lower body lift Lower body lift surgery Body lift Body lift surgery
Malar augmentation	Malar augmentation Cheek implant(s) Cheek implant surgery
Male breast reduction	Male breast reduction Male breast reduction surgery Gynecomastia surgery
Mastopexy	Mastopexy Breast lift Boob job Breast enhancement
Mentoplasty	Mentoplasty Chin lift Chin lift surgery Chin filler Chin augmentation
Otoplasty	Otoplasty Ear lift Ear surgery Ear lobe repair
Pectoral implant	Pectoral implant(s) Pec implants Pec surgery
Platysmaplasty	Platysmaplasty Neck lift Neck lift surgery
Rhinoplasty	Rhinoplasty Rhinoplasty surgery Nose job
Rhytidectomy	Rhytidectomy Face lift Face lift surgery
Thighplasty	Thighplasty Thigh lift Thigh lift surgery

Bivariate regression analysis was performed on 25 cosmetic plastic surgery procedures, including variations in search terms (Table 2). All analyses were completed using Stata, version 16.1 (StataCorp LLC, College Station, TX). Analysis of panel data was then applied to the aggregate trend in popularity to determine whether a statistically significant change in search volume occurred following the increase in user traffic of the Instagram platform.

**Table 2.** Bivariate Regression Results for Cosmetic Surgical Procedures

Procedure	Coefficient	Standard error	*z*-score	*P* > |*z*|	95% Coefficient interval
Abdominoplasty	0.004	0.030	0.13	.899	(−0.055, 0.063)
Blepharoplasty	0.170	0.028	6.14	.000	(0.116, 0.224)
Botox	0.365	0.029	12.74	.000	(0.309, 0.421)
Brachioplasty	0.616	0.035	17.74	.000	(0.548, 0.684)
Breast augmentation	−0.346	0.048	−7.16	.000	(−0.440, −0.251)
Breast implant removal	0.193	0.026	7.32	.000	(0.141, 0.244)
Breast reduction	0.106	0.034	5.75	.000	(0.053, 0.160)
Brow lift	0.203	0.022	9.26	.000	(0.160, 0.246)
Buccal fat removal	0.583	0.036	16.25	.000	(0.512, 0.653)
Butt lift	1.186	0.097	12.24	.000	(0.996, 1.375)
Calf augmentation	−0.076	0.019	−4.02	.000	(−0.112, −0.039)
Hair transplantation	−0.110	0.037	−2.94	.003	(−0.184, −0.037)
Lip augmentation	0.325	0.041	7.95	.000	(0.245, 0.405)
Lip reduction	0.063	0.028	2.22	.026	(0.007, 0.118)
Lower body lift	−0.011	0.035	−0.33	.741	(−0.080, 0.057)
Malar augmentation	−0.121	0.033	−3.70	.000	(−0.186, −0.057)
Male breast reduction	0.095	0.033	2.86	.004	(0.030, 0.159)
Mastopexy	0.085	0.028	−2.98	.003	(−0.141, −0.029)
Mentoplasty	0.395	0.047	8.40	.000	(0.303, 0.487)
Otoplasty	0.151	0.035	4.27	.000	(0.082, 0.220)
Pectoral implant	−0.059	0.036	−1.62	.104	(−0.129, 0.012)
Platysmaplasty	0.278	0.021	13.24	.000	(0.237, 0.319)
Rhinoplasty	0.093	0.025	3.79	.000	(0.045, 0.142)
Rhytidectomy	0.065	0.021	3.08	.002	(0.024, 0.106)
Thighplasty	0.117	0.034	3.41	.001	(0.050, 0.185)

## RESULTS

The analysis reported statistically significant differences in search volumes for plastic surgery procedures after April 2012. Blepharoplasty (*P* < .000), Botox (*P* < .000), brachioplasty (*P* < .000), breast implant removal (*P* < .000), breast reduction (*P* < .000), brow lift (*P* < .000), butt lift (*P* < .000), hair transplantation (*P* < .000), lip augmentation (*P* < .000), male breast surgery (*P* < .000), mastopexy (*P* < .000), mentoplasty (*P* < .000), otoplasty (*P* < .000), platysmaplasty (*P* < .000), rhinoplasty (*P* < .000), and thighplasty (*P* < .000) had statistically significant increases in search volume. However, Buccal fat removal (*P* = .003) showed a statistically significant decrease in search term volume. Coefficient, standard error, and *P*-values are all reported in Table 2. Search trends for the 25 plastic surgery procedures over 18 years are given in the Appendix.

## DISCUSSION

The new age of social media has brought on unique ways of disseminating medical, promotional, and educational information. Although online trends have been historically difficult to capture, the emergence of new tools like Google Trends has facilitated the analysis of trending topics through a comparison of search terms.^9^ Recently, the Google trends tool has been used to analyze changes in the interest for various cosmetic plastic surgical procedures as well as report evidence of an increase in above-the-shoulder procedures following an increase in virtual video meetings in the setting of the 2020 SARS-CoV2 pandemic.^7,10^ The literature demonstrates the utility of Google Trends in tracking interest in surgical procedures. In this study, we turn attention to the trend of different plastic surgical procedures with the emergence of Instagram, 1 of the most popular social media applications available to the public.

Our study aims to explore public interest in aesthetic procedures before and after the Instagram platform was released in 2012. Through hashtag links, search terms, and dedicated profiles, Instagram has been a core medium for the spread of medical procedures and information. The current literature highlights the increased use of social media to share and connect individuals who are interested or who require procedures in specialties such as general surgery, craniofacial surgery, gynecological surgery, orthopedic surgery, and neurosurgery.^11–15^ As of recent date, the spread of plastic surgery procedures through Instagram profiles, hashtags, and search terms has gained popularity as a valid channel for promotion. In this study, we hypothesize that as a result of increased medical marketing on Instagram, there is an increase in public interest in plastic surgery elective procedures.

Statistically significant increases in search volumes were seen after April 2012, which marked the release of the Instagram platform for public use. Plastic surgery procedures showing increased volume of searches included abdominoplasty, blepharoplasty, Botox, brachioplasty, breast implant removal, breast reduction, brow lift, butt lift, hair transplantation, lip augmentation, male breast surgery, mastopexy, mentoplasty, otoplasty, platysmaplasty, rhinoplasty, and thighplasty. This increase in search trend is shown to correlate with increased popularity.^9^ Conversely, buccal fat removal had a decrease in search volume, indicating a decrease in popularity. Through these findings, it is shown that with the emergence of Instagram, there has been an increased public interest in plastic surgical procedures.

The role of social media in the plastic surgery space has been a subject of study and discussion for many researchers.^16–18^ Since their inception, social media platforms like Instagram, Twitter (San Francisco, CA), and most recently, TikTok (Culver City, CA), have been used for online discourse in medicine spanning a vast number of topics. By searching for only a specific term, Instagram has facilitated the connection of information with individuals who seek to learn more about these subjects. With the focus on plastic surgery, individuals can inquire about potential elective procedures directly from surgeons who promote their work.^18^ With the gradual advent of a post-COVID era, a further push toward online connection is expected, leading to an increase in the search for information that was previously obtained through in-office consultation visits that may be limited by a number of factors, including travel, cost, availability, and access. Patients can also readily compare procedures that are offered at their fingertips, actively comparing techniques both surgical and nonsurgical in nature, institutions, and surgeons from virtually anywhere around the world.^1^

In addition to procedural information, social media platforms like Instagram provide an opportunity for engagement and branding for aesthetic plastic surgeons. Surgeons are able to utilize the platform to reach potential clients and consumers through a demonstration of their expertise by making before and after comparisons or connecting directly through personal messaging consultations.^5^ In turn, this could lead to the expansion of a plastic surgeon's network, which can be of particular use for newer surgeons who have yet to establish a substantial clientele. With the significant effect that Instagram has on the popularity of elective procedures seen in this study, its use for the promotion of various practices could, in theory, be expected as well.^16^

The statistically significant increase in search volumes seen in this study also correlates with an increased demand for plastic surgery procedures. Before the advent of social media, patients located physicians by word of mouth and recommendations from trusted individuals. As Instagram and other social media platforms have gained popularity, these patients have been able to shift from a word-of-mouth referral system to a more complex internet search system. It is possible that patients are utilizing internet searches to gain preliminary information about their providers and specific procedures instead of asking others. Additionally, in recent studies, researchers show the influence of social media on various cosmetic procedures and changes in demand for noninvasive procedures compared with invasive ones. In their study, Hopkins et al found that there was an increase in noninvasive trademarked procedures through Google Trends—eg, CoolSculpting (Allergan, Irvine, CA), Juvederm (Allergan, Irvine, CA), Kybella (Allergan, Irvine, CA)—compared with the categories of procedures considered to be traditional options. Although in this study, we focused on the increase in noninvasive cosmetic procedures, we similarly showed evidence of an increase in cosmetic procedural trends as we enter the age of social media.^19^

In the last few years, there has been an increase in the utilization of online connectivity to facilitate the integration of platforms in the setting of limited travel and in-person meetings. The SARS-CoV2 pandemic has created an opportunity for the use of platforms like Instagram to educate and inform not only the general public but also those in the medical field. The beauty of social media is that we can not only increase patient awareness and advocacy but can also use the platform to inspire prospective students in the field of plastic surgery. These last few years have increased the popularity of visual educational resources and online virtual curriculum spread primarily through Instagram.^20,21^ Initiatives like “PRS MedEd” use virtual webinar lecture series as supplemental courses for students interested in learning about plastic surgery topics.^22^ Instagram has eased the ability to connect with students, residents, and established plastic surgeons, with many researchers in their studies identifying useful ways to integrate the platform into various practice settings.^5^

Apart from the facilitation of connectivity among surgeons, residents, students, and the general population, 1 of the greatest utilizations that the authors of this study find can be of benefit is the use of online platforms to increase transparency in the field. Unfortunately, misinformation can also be spread with ease throughout Instagram, Twitter, and other popular platforms. Engaging with discussion on Instagram can provide an expert voice from an educated and trained background to debunk myths and misconceptions and speak against misinformation in the field. However, the use of these platforms may still present a balancing act in which curated content can also be misleading or may not be as comprehensive to the entirety of the field of plastic surgery.

Google Trends is a publicly accessible tool that has been shown to be a valid measure of interest in the population. The main limitation to the use of Google Trends is its inability to provide absolute search term numbers. Its ability to identify only relative values of search terms may skew the data, especially when considering confounding variables—including increased public discussion of *viral* aesthetic plastic surgical procedures popularized by celebrities and people of influence. The use of synonyms for plastic surgery procedures included in this study may also not be exhaustive for possible terms. Terms like buccal fat removal or bichectomy may shift in popularity as more colloquial terms emerge throughout the years making that data difficult to capture. These trends may also reflect changes in procedures that plastic surgeons may recommend to their patients based on emerging literature.^23,24^ Furthermore, these limitations can influence the type and scope of statistical analyses able to be performed. In order to mitigate these limitations, recent literature that sets forth guidelines and frameworks for the use of Google Trends has emerged.^25^ Lastly, no trend on social media that considers various aspects of society as does changes in popularity, new discoveries, and aging of younger generations occurs in isolation. There are many factors that could contribute to the increase of aesthetic surgeries that may affect our findings including trends on other forms of media—television and written journalism—or the effect of celebrity influence and news trends. Future studies are needed to establish a causal relationship between social media use and interest in plastic and aesthetic surgery procedures.

## CONCLUSIONS

Instagram as a visual platform is uniquely suited for the field of aesthetic plastic surgery. As a media that is largely centered around photography and video consumption, Instagram eases the spread of available aesthetic procedures through the promotion of live models and influencers that share their experiences and results. In our study, we observed an increase in public interest in both surgical and nonsurgical aesthetic procedures after the emergence of Instagram in April 2012, indicating its potential use for connectivity within the plastic surgery world. As more plastic surgeons engage in social media marketing, understanding and utilizing these trends could help increase practice awareness on potential patients, information dissemination, social media engagement, and medical education in the field of plastic surgery.

## Supplementary Material

ojad004_Supplementary_Data
