# Supplementary material for: #PRS: A Study of Plastic Surgery Trends With the Rise of Instagram
Source: Aesthet Surg J Open Forum. 2023 Jan 11;5:ojad004. doi: 10.1093/asjof/ojad004 (PMC9915973; doi:10.1093/asjof/ojad004)
Supplement: ojad004_Supplementary_Data [file ojad004_supplementary_data.docx]

**Appendix:** Trend Lines of Procedure Type
